# Moderating Effect of Psychological Resilience on Functional Impairment in Relation to Subsequent Depression

**DOI:** 10.1093/geroni/igaa057.1566

**Published:** 2020-12-16

**Authors:** Shinae Choi, Eun Ha Namkung

**Affiliations:** 1 The University of Alabama, Tuscaloosa, Alabama, United States; 2 Brandeis University, Waltham, Massachusetts, United States

## Abstract

The growing prevalence of functional impairment is a serious concern due to its relation to decreased quality of life in later life. Guided by the social convoy model and the stress process model, the present study investigated whether psychological resilience, particularly optimism and mastery moderated an association between functional impairment and subsequent depressive symptoms in later life. This study used data derived from two population-based national studies in the United States: 2012 and 2016 waves of the Health and Retirement Study (N = 5,035) and 2004 and 2013 waves of the Midlife in the United States (N = 2,476). Ordinary least squares regression was used to estimate the impact of optimism and mastery, respectively, on the associations between functional impairment (baseline measure at wave(t-1), changes over the study period from wave(t-1) to wave(t)) on subsequent changes in depressive symptoms. Across both studies, we found that having and developing functional impairment are related to increased number of depressive symptoms. Optimism independently predicted decreased depressive symptoms over the study periods and buffered the negative effects of functional impairment on depressive symptoms across the two studies. Specifically, the mitigating effects of optimism on depressive symptoms were greater for those with more numbers of functional limitations. The findings suggest that psychological resilience plays a key role in decreasing depressive symptoms, especially for midlife and older adults with functional impairment. The results also demonstrate the importance of examining both optimism and mastery when investigating psychological resilience and emotional well-being in older adults.

